# Neuroplacentology in congenital heart disease: placental connections to neurodevelopmental outcomes

**DOI:** 10.1038/s41390-021-01521-7

**Published:** 2021-04-16

**Authors:** Rachel L. Leon, Imran N. Mir, Christina L. Herrera, Kavita Sharma, Catherine Y. Spong, Diane M. Twickler, Lina F. Chalak

**Affiliations:** 1 Department of Pediatrics, University of Texas Southwestern Medical Center, Dallas, TX USA; 2 Department of Obstetrics and Gynecology, University of Texas Southwestern Medical Center, Dallas, TX USA; 3 Department of Radiology, University of Texas Southwestern Medical Center, Parkland Health and Hospital Systems, Dallas, TX USA

## Abstract

**Abstract:**

Children with congenital heart disease (CHD) are living longer due to effective medical and surgical management. However, the majority have neurodevelopmental delays or disorders. The role of the placenta in fetal brain development is unclear and is the focus of an emerging field known as neuroplacentology. In this review, we summarize neurodevelopmental outcomes in CHD and their brain imaging correlates both in utero and postnatally. We review differences in the structure and function of the placenta in pregnancies complicated by fetal CHD and introduce the concept of a placental inefficiency phenotype that occurs in severe forms of fetal CHD, characterized by a myriad of pathologies. We propose that in CHD placental dysfunction contributes to decreased fetal cerebral oxygen delivery resulting in poor brain growth, brain abnormalities, and impaired neurodevelopment. We conclude the review with key areas for future research in neuroplacentology in the fetal CHD population, including (1) differences in structure and function of the CHD placenta, (2) modifiable and nonmodifiable factors that impact the hemodynamic balance between placental and cerebral circulations, (3) interventions to improve placental function and protect brain development in utero, and (4) the role of genetic and epigenetic influences on the placenta–heart–brain connection.

**Impact:**

- Neuroplacentology seeks to understand placental connections to fetal brain development.
- In fetuses with CHD, brain growth abnormalities begin in utero.
- Placental microstructure as well as perfusion and function are abnormal in fetal CHD.

## Introduction

Congenital heart disease (CHD) affects an estimated 40,000 neonates annually in the United States, which is approximately 1% of all live births.^1,2^ Many of the severe forms of CHD require surgical repair during the neonatal period or later in infancy. These types of CHD are associated with an increased risk of neurodevelopmental delays and disorders.^3^ Now that survival in neonates and children with CHD has increased significantly with improving surgical techniques as well as medical treatment options, there is a new focus on optimizing neurodevelopmental outcomes.^4^

Some preliminary studies suggest that the placenta in pregnancies complicated by CHD has a higher rate of both structural and functional abnormalities.^5–7^ The growing field known as neuroplacentology seeks to understand the influence of this vital organ on fetal brain development.^8^ Its impact involves both de novo synthesis of key neurotransmitters^9^ and hormones,^10^ as well as the maintenance of a vital hemodynamic balance to ensure adequate blood flow and oxygen delivery to the developing brain.^11^ In fetuses with CHD, an imbalance in the prenatal hemodynamic relationship may contribute to preoperative brain abnormalities and to the neurodevelopmental impairments in the CHD population, in addition to the impact of events in the neonatal period and beyond. Those events vary by cardiac lesion, but for many neonates include a period of postnatal hypoxia, intubation and exposure to volatile anesthetic agents, pain and analgesic medications, cardiac catheterization, cardiac surgery that may include cardiopulmonary bypass, deep hypothermic circulatory arrest, postoperative recovery, and hospitalization.

Despite these risk factors and high rates of neurodevelopmental delays and impairments in children with CHD, studies of school-age children with cardiac interventions in the first year of life have not found significant associations with perioperative factors and developmental or educational outcomes.^12,13^ The idea of a prenatal origin of brain maldevelopment in children with CHD warrants further exploration. The role of placental hemodynamics in fetal brain development is unclear, and the currently available non-invasive tools, such as Doppler ultrasound, advanced magnetic resonance imaging (MRI) techniques, and placental pathologic examination, to study the placenta are under-utilized.^14,15^

This article reviews the literature on neurodevelopmental outcomes in CHD patients including data suggesting neurodevelopmental impairments may arise from disruptions to brain development prenatally. Specifically, we review brain imaging abnormalities in those with CHD, including the increased prevalence of abnormalities such as delayed maturation, decreased global and regional brain volumes, and white matter injury on fetal brain MRI. We examine the link between aberrant fetal brain development and abnormalities in placental structure and function. We hypothesize that disruptions in placental hemodynamics may have subtle deleterious effects on fetal brain development in those with CHD. We conclude this review with future research directions and considerations for the clinical care of the CHD population.

## Neurodevelopment in patients with CHD

### Neurodevelopmental outcomes in childhood and beyond

Children with CHD are significantly more likely to experience developmental delays and disorders compared to the general population.^12,16^ Prevalence and severity of developmental delays and disorders in children with CHD are directly related to severity of their heart disease,^3^ and these delays and disorders span all domains of development.^13,17–24^ Neurodevelopmental delays and disorders can be diagnosed as early as infancy and are particularly common in neonates with CHD and comorbid conditions, such as prematurity.^12,25^ As many as 15% of preschool-age children with CHD who require surgery score in the at-risk or clinically significant range on scales of pervasive developmental problems.^26^ In addition, they have higher rates of attention and learning problems^27^ and high rates of motor deficits.^28^ Sequelae of neurodevelopmental delays and disorders persist into school age and beyond. In a subgroup of infants with transposition of the great arteries (TGA), the Boston Circulatory Arrest Trial showed that 19–22% of their cohort of 155 children had problem behaviors at age 8 years according to parental and teacher assessments.^29^ In children who underwent staged palliation for hypoplastic left heart syndrome (HLHS), as many as one-third require remedial education services during elementary school and cognitive testing in a small cohort demonstrated intellectual disability in 18%.^20^

Likewise, in adolescence, CHD patients have a significantly higher rate of memory deficits compared to healthy peers,^30^ as well as an increased need for remedial education services.^22^ In adulthood, CHD survivors have lower educational and occupational levels compared to healthy control groups^22^ and significantly lower scores on validated assessments of quality of life.^31^ Psychiatric morbidity occurs at a higher rate in these adults as well, spanning from major depressive disorder and panic disorder^32^ to obsessive-compulsive symptoms and psychosis.^33^ One leading expert in the field contends that neurodevelopmental challenges remain the most prevalent long-term adverse consequence of CHD and its treatment and are more common than all cardiac sequelae of their condition.^34^ The challenges that children with CHD face have led to specific recommendations by the American Heart Association in 2014 for the screening, diagnosis, and management of neurodevelopmental delays in this population that focus on risk stratification, enhanced screening into adolescence, and interventional services.^3^

### Multifactorial etiology of neurodevelopmental impairments

The multifactorial influences on neurodevelopment in the CHD population are clear, yet the relative importance of prenatal, surgical, and post-surgical factors remains unknown. Although some of the increased incidence of neurodevelopmental disorders are related to underlying genetic conditions, only an estimated 23% of CHD patients have aneuploidies and copy number variations.^35–37^ Nevertheless, severe forms of CHD—particularly those that necessitate surgical intervention in the neonatal period—impart multiple potential causative factors for developmental problems and disrupted brain development. These factors include prolonged postnatal hypoxia while awaiting surgical repair,^38,39^ volatile anesthetic exposure,^40,41^ cardiopulmonary bypass,^42^ postoperative recovery with its complications, and prolonged hospitalization. In addition, new data have demonstrated that exposure to plastics may also contribute to impaired neurodevelopment in this population.^43^ Other reports have found significant associations between cardiopulmonary bypass with regional cerebral perfusion, lower intraoperative cerebral hemoglobin oxygen saturation during the period of myocardial ischemia, and postoperative brain injury.^44^ The duration on cardiopulmonary bypass, use of deep hypothermic circulatory arrest, and elevated postoperative lactate levels, as well as preoperative white matter injury, have been correlated with postoperative white matter injury in a multivariable model that prospectively enrolled 147 neonates with CHD.^45^

Many conflicting reports exist in the literature with most studies confounded by heterogeneous CHD populations and/or small sample sizes. In one study of 109 school-aged children with CHD requiring surgery in the neonatal period, investigators found no association between perioperative events including cardiac diagnosis, cardiopulmonary bypass time, and incidence of postoperative cardiac arrest or seizures, with the use of remedial school services or diagnosis of ADHD.^13^ Similar findings were confirmed by Lawley et al. in a population-based linkage study of school-age outcomes in 260 children with CHD in Australia.^12^ Another study found correlation between Bayley Scales of Infant Development III (BSIDIII) at 24 months and many factors of the home environment, preoperative health, and operative factors, among others. However, in their multivariable analysis, intraoperative factors were not found to be independently associated with BSIDIII scores.^46^ In a cohort of 328 children with single-ventricle physiology from the Pediatric Heart Network, Wolfe et al. found no relationship between peripheral oxygen saturations following state I and stage II palliative surgeries and neurodevelopmental outcomes at 14 months, even when controlling for relevant covariates (any SpO_2_ <80% relative risk 2.25 [95% confidence interval (CI) −1.55, 6.06], *p* = 0.247).^47^

The impact of anesthetic exposure is impossible to study in isolation; however, studies comparing neonates undergoing non-cardiac surgery versus cardiac surgery indicate that neurodevelopmental outcomes are worse for children with CHD.^48^ This may be confounded by the fact that many with CHD undergo multiple surgeries in childhood, as the number of surgical procedures has been associated with progressively deleterious impact on neurodevelopment in a large birth cohort study in Japan.^49^ Together, these data suggest that the etiology of neurodevelopmental outcomes in children with CHD are complex, and multifactorial, depending upon factors both within and outside the surgical and postoperative periods.

## Structural and functional brain abnormalities

### Postnatal brain imaging

As expected from their neurodevelopmental impairments, children with CHD have an increased incidence of brain abnormalities in imaging studies.^44,50–62^ In a systematic review and meta-analysis that included 221 cases of CHD, Khalil and colleagues reported the prevalence of brain lesions on MRI in TGA to be 34%, in left-sided heart lesions 49%, and in mixed/unspecified heart lesions 46%.^63^ White matter injury is currently thought to contribute most to overall neurodevelopmental outcomes.^64^ In a prospective, longitudinal study of 104 infants with single-ventricle physiology or TGA, Peyvandi and colleagues found a significant association with moderate-to-severe white matter injury with impaired neurodevelopment at 30 months of age.^65^ The timing of injury is difficult to discern with the incidence of postoperative brain injury in infants undergoing open heart surgery estimated at 34%,^57^ indicating a role for intraoperative factors.

However, neonates with complex CHD have abnormal brain structure and maturation even prior to corrective heart surgeries. Using MRI, MR spectroscopy, and diffusion tensor imaging in a cohort of neonates with TGA and single-ventricle physiology imaged between 4 and 9 days of life, Miller et al. demonstrated significant alterations in MR spectroscopy including decreased *N*-acetylaspartate-to-choline ratio and increased ratio of lactate to choline indicative of a delay in brain maturation.^54^ The estimated delay in maturation in a small study of term neonates with HLHS and TGA was reported as approximately 1 month based on a maturation scoring system that evaluates myelination, cortical in folding, involution of glial cell migration bands, and presence of germinal matrix tissue.^66^

White matter injury is thought to be one of the most common brain abnormalities in newborns with severe forms of CHD,^52,54,61^ but increasingly, attention is being placed on cortical gray matter and functional connectivity.^60,62,67^ In the cohort imaged by Miller and colleagues, white matter injury was found in 30% to as much as 69% of preoperative infants with CHD in the first week of life.^53,54^ White matter injury in preoperative neonates with CHD has been shown to have predilection for anterior and posterior locations, rather than the central white matter injury seen in preterm infants.^57^ Supporting the evidence of highly affected anterior brain regions, Ortinau et al. demonstrated significantly smaller frontal lobe volumes in their series of 67 neonates with preoperative complex CHD (defined as those requiring surgery in first 2 months of life) imaged, on average, by the eighth postnatal day.^52^

### Fetal brain imaging

An increasing number of fetal imaging studies show specific abnormalities in brain growth and maturation that begins in utero for fetuses with CHD. In series ranging from 5 fetuses imaged every 4 weeks during the second half of gestation^68^ to cohorts of 73 fetuses with CHD imaged once,^50^ several research groups have reported that fetuses with CHD have a smaller brain volume compared to healthy counterparts.^50,68–71^ As demonstrated postnatally, specific brain regions are more vulnerable to lagging growth in fetuses with CHD, particularly in the third trimester, a period of rapid cortical development and expansion of white matter.^72^ Paladini et al. recently reported impaired frontal lobe growth that plateaus around 30 weeks gestation in a cohort of 101 fetuses with CHD compared to >400 healthy controls.^73^ Others have demonstrated that in single-ventricle physiology CHD fetuses, poor brain growth in pregnancy is driven by reduced growth of gray matter structures, including the cortical plate, deep gray matter, and the cerebellum.^74^ Those fetuses with antegrade aortic flow compared to ductus arteriosus-dependent systemic flow has not been shown to correlate with the lagging head growth in fetuses with CHD;^75^ rather, adaptive cerebral vasodilation as measured by the cerebroplacental ratio of pulsatility indices appears to be linked to smaller head size.^76^

### Pathophysiology of prenatal brain injury

Most neonates with CHD have lower oxygen saturations postnatally and some experts have suspected a prenatal origin of neonatal brain abnormalities.^11,77,78^ Disrupted brain development in fetal CHD mirrors the pathology described in animal models of chronic hypoxia, specifically, white matter volume loss and decreased brain growth.^79^ Using phase-contrast MRI and T2 mapping, one imaging study showed significant decreases in fetal cerebral oxygen consumption in complex CHD fetuses compared to controls (2.7 ± 1.2 mL/min/kg in CHD group versus 4.0 ± 1.2 mL/min/kg in healthy controls; *p* = 0.0001).^11^ These findings support the hypothesis that adaptive cerebral vasodilation is inadequate to ensure normal brain development, thus questioning the notion of “brain sparing,” which has been used to describe the fetal cerebral vasodilatory response to impaired cerebral blood flow and oxygen delivery. In fact, in studies of fetuses with CHD, vasodilatation of cerebral vasculature by Doppler ultrasound has been demonstrated in multiple cerebral arteries^80,81^ and is described in multiples studies of fetuses with HLHS.^82–84^

The effects of chronic hypoxia on brain development have been shown to depend on timing of initiation of the insult. Earlier initiation of hypoxia is associated with more widespread white matter injury, as described in fetal sheep.^85^ Sheep exposed to hypoxia later in gestation show reduced myelin, neuronal apoptosis in areas of cortex, and decreased numbers of mature oligodendrocytes.^85^ The suspected etiology of these injuries lies in the effects of chronic hypoxia on the sensitive population of cells known as the premyelinating oligodendrocytes, which arise from the subventricular zone and serve to myelinate neuronal axons.^86,87^ Back and colleagues have demonstrated a key developmental window of susceptibility for these cell populations in an animal model of hypoxic–ischemic injury^86^ correlating to approximately 23–32 weeks gestation in human fetuses, which coincides with emergence of lagging head growth and perfusion disturbances in CHD fetuses.^50^

## Role of the placenta

**Fig. 1:**
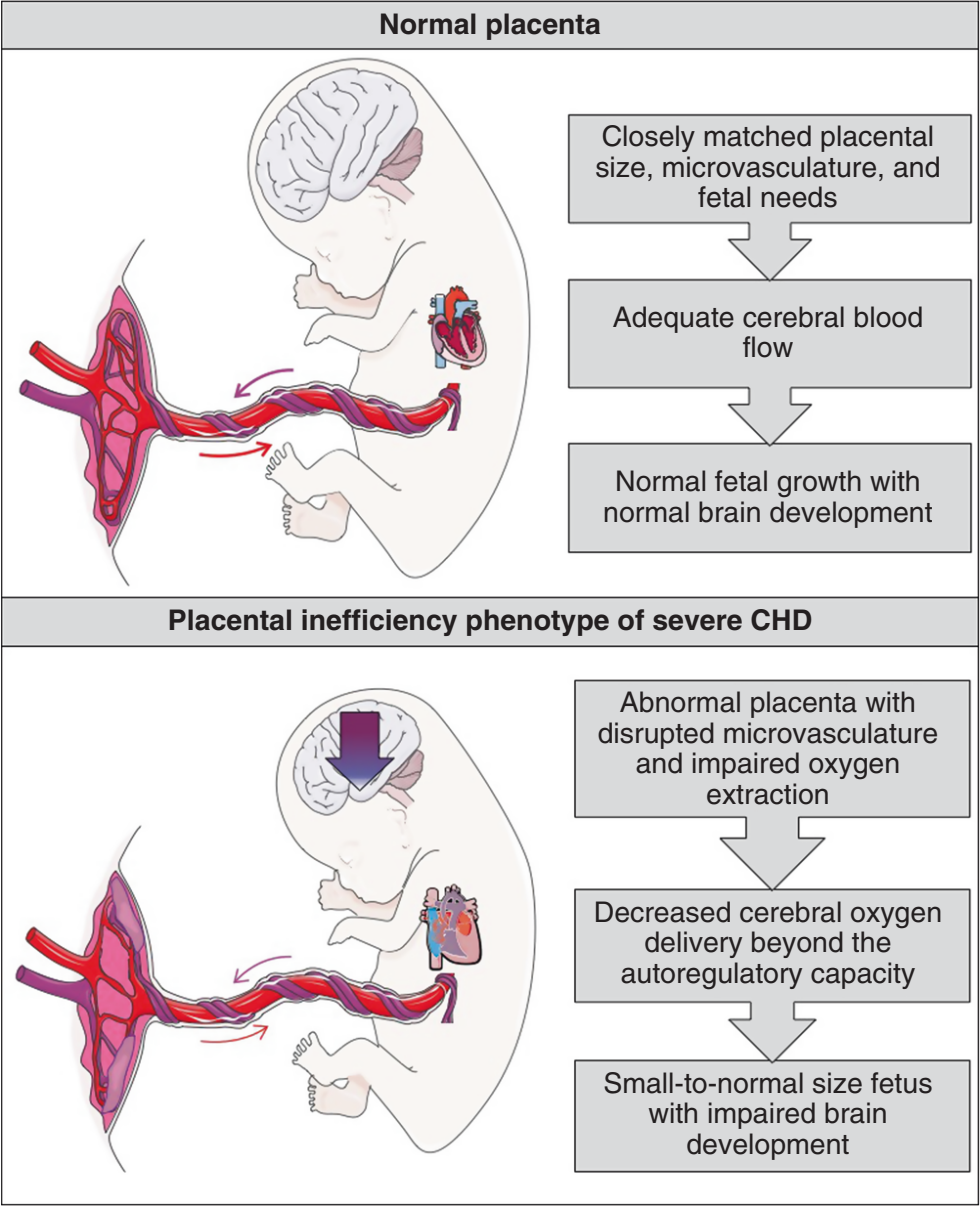
Normal placenta characterized by closely matched size and function to fetal needs compared to the inefficiency phenotype of fetal CHD. This placenta is characterized by an inefficient function with vascular immaturity and a myriad of placental pathologic lesions, which leads to decreased cerebral blood flow beyond the autoregulatory capacity of the fetus with CHD, resulting in a small-to-normal size fetus with impaired brain development. CHD congenital heart disease.

It should come as no surprise that the placenta plays an important role in fetal brain development. At term, the placenta receives ~40% of fetal cardiac output and is the largest fetal organ. Neonates with CHD and superimposed placental dysfunction including gestational hypertension, pre-eclampsia, preterm birth, and growth restriction demonstrate higher mortality and increased hospital length of stay than their counterparts with a healthy placenta.^88^ There is growing evidence that the placenta in pregnancies complicated by fetal CHD may have morphologic and functional changes, but the pathophysiologic mechanisms linking aberrant placental structure and function to fetal brain abnormalities remains unknown. The fetal heart and placenta are both vascular organs of fetal origin, indicating that placental vasculature may also be disrupted in fetal CHD, although there is conflicting evidence in the literature. In some imaging studies, the placenta in fetal CHD has a larger volume than expected for the fetal size^5,6^ along with pathologic evidence of reduced arborization of the fetal villi.^89^ This data suggests a different structural phenotype in CHD pregnancies that is distinct. We propose a new framework in considering the role of the placenta in fetal CHD, namely, that the placenta in these pregnancies is functionally inefficient and structurally impaired (Fig. 1). We postulate that, in some pregnancies complicated by fetal CHD, the fetus perfuses an immature placental microvasculature that may be disrupted by multiple pathologies, thus preventing maximal oxygenation of fetal blood, leading to lower oxygen saturation of blood coming from the placenta. In these fetuses, this ultimately results in lower cerebral oxygen delivery, poor brain growth, and impaired neurodevelopment. Functional imaging of the placenta as well as many of the studies on histologic examination of the CHD placenta support this new placental classification and will be reviewed in the sections that follow.

### Functional placental imaging

Functional placental imaging indicates a possible role for placental malfunction in the brain abnormalities seen in fetuses with CHD. Advanced MR imaging has expanded our understanding of the placenta by allowing volumetric growth curves throughout gestation,^90,91^ quantification of placental blood flow from the maternal compartment,^7^ and textural analysis as the placenta matures.^14^ These techniques have provided insights into placental function in a diverse range of pregnancy-related disease states, including fetal CHD. In a cohort of women pregnant with fetuses diagnosed with either biventricular or single-ventricle physiology CHD, You et al. used blood oxygen-level-dependent MRI (BOLD MRI) to show differential changes in the placental and fetal brain BOLD signal with maternal hyperoxygenation.^92^ Fetuses with single-ventricle physiology CHD had a significantly greater increase in BOLD signal in the placenta compared to controls and compared to pregnancies of fetuses with biventricular CHD (mean Delta R2* 1.9 s^−1^ ± 0.2 for single-ventricle CHD, 1.0 s^−1^ ± 0.3 for biventricular CHD, and 1.3 s^−1^ ± 0.1 for controls; *p* < 0.01). The fetal brain BOLD signal did not increase from baseline with maternal hyperoxygenation in healthy controls or those with fetal biventricular CHD, but in single-ventricle fetal CHD, fetal brain BOLD signal increased quickly with maternal hyperoxygenation and remained higher even after discontinuation of maternal oxygen administration.^92^

Corroborating evidence using Doppler ultrasound of middle cerebral artery pulsatility index was demonstrated by Szwast et al. where maternal hyperoxygenation increased middle cerebral artery pulsatility index in those with fetal HLHS.^93^ Although suggestive of an oxygen response in cerebral vascular resistance, this study lacked healthy control comparisons. Umbilical venous volume flow has also been shown to be decreased in mid-gestation in fetuses with single-ventricle CHD compared to controls [96.5 ± 24.3 mL/min/kg in single-ventricle CHD (*n* = 24) versus 118.5 ± 30.5 mL/min/kg in controls (*n* = 141); *p* = 0.001] but does not represent a statistically significantly smaller portion of estimated total cardiac output (23.9 ± 9.3% in single-ventricle CHD compared to 27.2 ± 8.8% in controls; *p* = 0.125).^94^ Using phase-contrast MRI and T2 mapping, Sun et al. have shown that fetuses with severe forms of CHD have a lower oxygen saturation in the umbilical vein compared to controls and an overall 13% reduction in brain volume in fetal CHD subjects.^11^ In sophisticated studies of 30 late-gestation fetuses with severe forms of CHD and 30 healthy controls, they mapped the decreased oxygen saturation from the umbilical vein (73 ± 9% in CHD group versus 79 ± 5% in healthy controls; *p* = 0.004) to the ascending aorta and showed decreased cerebral oxygen consumption in the CHD cohort as well as reduced total brain volume.^11^ This data suggests that, in severe forms of CHD, the cerebral autoregulation plateau is exceeded and cerebral oxygenation becomes compromised in some fetuses with CHD. To compensate for this decreased oxygen delivery, one would expect a physiologic adaptation allowing for greater oxygen extraction, but there was no increase in cerebral oxygen extraction in the CHD group (32 ± 20% in CHD group versus 34 ± 8% in healthy controls; *p* = 0.53).^11^ These values are most pronounced in their fetuses with single-ventricle physiology, highlighting the need for replication of these studies in a larger cohort of specific CHD physiology. Particularly of interest would be comparisons between fetuses with antegrade versus retrograde flow in the aortic isthmus.

The interpretation of this data is complex but suggests the potential of a dynamic relationship between the fetal brain, heart, and placenta in the setting of severe CHD. In the BOLD hyperoxygenation imaging study, the increase in placental oxygenation signal in fetuses with single-ventricle CHD compared to both fetuses with two-ventricle CHD and healthy controls suggests an ability to increase oxygen saturation perhaps secondary to structural or physiologic differences in the placenta or a greater deficit in its oxygen reservoir. The greater deficit could be explained by either decreased placental uptake from maternal circulation or greater extraction by the fetal circulation. The possibility of a baseline deficient placental uptake of oxygen from maternal circulation driving the increase in placental BOLD signal in the setting of maternal hyperoxygenation is supported by a recent study showing uteroplacental malperfusion with significantly higher uterine artery pulsatility indices in pregnancies complicated by fetal CHD compared to healthy controls (0.90 multiples of the median (MoM), *n* = 153 versus 0.83 MoM, *n* = 658; *p* = 0.006).^95^ This preliminary data requires further studies to corroborate this evidence and determine a mechanistic explanation. The finding by Sun et al. of decreased umbilical vein oxygen saturation in fetal CHD suggests that the placenta may play a role in decreased brain growth through impaired cerebral oxygen delivery in some fetuses with CHD. Unfortunately, these studies do not include histopathology of placental specimens after delivery, thus it remains unknown whether changes in oxygenation are related to structural and microvascular pathology of the placenta.

### Placental histopathology in fetal CHD

Histopathologic studies of the placenta from pregnancies complicated by fetal CHD support the notion of placental dysfunction, but these studies have significant limitations. Due to the infrequency of some CHD diagnoses necessitating heterogeneous grouping of CHD subtypes and the inconsistency in performing placental pathologic examination in fetal CHD, these studies lead to mixed conclusions. Rychik et al. reported on placental pathology in 120 cases of CHD with groups of similar heart lesions grouped for subset analysis. Their primary findings in the total CHD group were thrombosis in 41% of placentas, infarction in 17%, chorangiosis in 18%, and hypomature villi in 15%.^96^ Unfortunately, this study did not examine a control group of placental pathology, thus leading to concerns about relative frequency in that center of the above findings. Likewise, maternal factors that impact placental pathology such as the presence of diabetes and gravida status were not included. Other reports have shown an increased incidence of abnormal cord insertion in all forms of fetal CHD, and fetuses with TGA have been shown to have the greatest number of placental abnormalities.^5^

There is mixed support from histopathologic studies for a placental inefficiency phenotype, and likely this phenotype pertains to the most severe forms of CHD. In a study of placentas from fetuses with HLHS, Jones et al. found reduced placental weight as well as reduced birth weight in most of the cohort.^89^ Placental-to-birth weight ratios were not calculated, but histologically, the placentas from HLHS patients appeared immature. Specifically, placentas showed increased syncytial nuclear aggregates, indicating failed branching of the villous tree.^89^ This was supported by the finding of decreased terminal villi. Interestingly, there was increased leptin expression in HLHS placenta, thought to be an attempt to compensate for the vascular immaturity.^89^ Leptin is produced by the placenta and serves as a pro-angiogenic hormone with leptin levels directly correlating with placental weight.^97^ Further studies showed a similar vascular disturbance in placentas from fetuses with TGA but without reduction in the birth weight-to-placental weight ratio.^98^ In the study by Albalawi et al., the placental-to-birth weight ratios were not different from controls but were significantly higher than those reported by Rychik et al., highlighting the imprecision of this metric due to factors, such as placental drain time and membrane trimming. However, in fetuses with CHD who were growth restricted, Albalawi et al. have reported an increased placental-to-birth weight ratio.^5^

## Shared developmental pathways

The placenta, heart, and brain share several key developmental pathways that, when disrupted, may explain some of the coinciding pathology in these organ systems. Specific genes and pathways that have been studied most include those involving angiogenesis, folic acid, and Wnt/planar cell polarity signaling, among others. These data have recently been reviewed^99–101^ and a full discussion is beyond the scope of this review. Studies have shown mixed results regarding the impact of these shared pathways, highlighting the fact that the multifactorial nature of neurodevelopmental outcomes in CHD remains incompletely understood.

Regarding angiogenic pathways, damaging variants in genes associated with promotion of angiogenesis were recently found to be present in 55% of a population of 133 neonates with complex CHD; but in this well-controlled study, a similar degree of damaging variants were also present in patients without CHD.^102,103^ Vascular endothelial growth factor (VEGF) has been shown to play a significant role in both cardiovascular, placental, and brain development. In animal models, VEGF is a major regulator in heart formation and its haploinsufficiency^104^ or overexpression^105^ both lead to embryonic lethality due to heart defects. Placental growth factor shares significant amino acid homology with VEGR and both bind the Flt-1 receptor to promote both vasculogenesis and angiogenesis. In the brain, neuropilin receptors bind VEGF and play a role in neural vascularization as well as heart development.^106^

Pathways utilizing folic acid have long been suspected to play a role in CHD and a recent case–control study from China built on the accumulating evidence from prior investigations supporting the role of folic acid supplementation in reducing the risk of fetal CHD.^107–111^ Qu et al. showed that women taking at least 0.4 mg of folic acid daily in the first trimester of pregnancy (with or without concurrent multivitamin use) had a significant reduction in the adjusted odds ratio (aOR) of any form of CHD [aOR 0.69; 95% CI 0.62–0.76 (*n* = 928 CHD, *n* = 949 Controls)], and lower aORs of most of the specific subtypes of CHD examined, although the sample sizes were likely inadequate for meaningful subgroup analyses.^112^ Most studies in folic acid supplementation are limited by their retrospective nature, possible role of recall bias, and lack of maternal blood folate levels to establish a causal relationship between folic acid intake and risk of CHD. Investigations that have examined maternal folate levels in relation to CHD risk have not found correlations,^113–115^ but this may be related to differential regulation of folate uptake and metabolism. Specific polymorphisms of multiple genes related to folate metabolism have been shown to correlate with CHD risk.^116–119^

The Wnt/planar cell polarity signaling pathway is recognized as essential in the development of multiple organ systems, including the heart from early tube formation to remodeling of outflow tracts.^120–122^ Wnt/planar cell polarity signaling has also been linked to brain development and specifically plays a role in neuronal migration, axonal sprouting, and disorders of these processes.^123^ This rapidly expanding field will undoubtedly provide greater understanding of the placenta–heart–brain connections and represents an important area for future investigations.

## Future directions in neuroplacentology

**Fig. 2:**
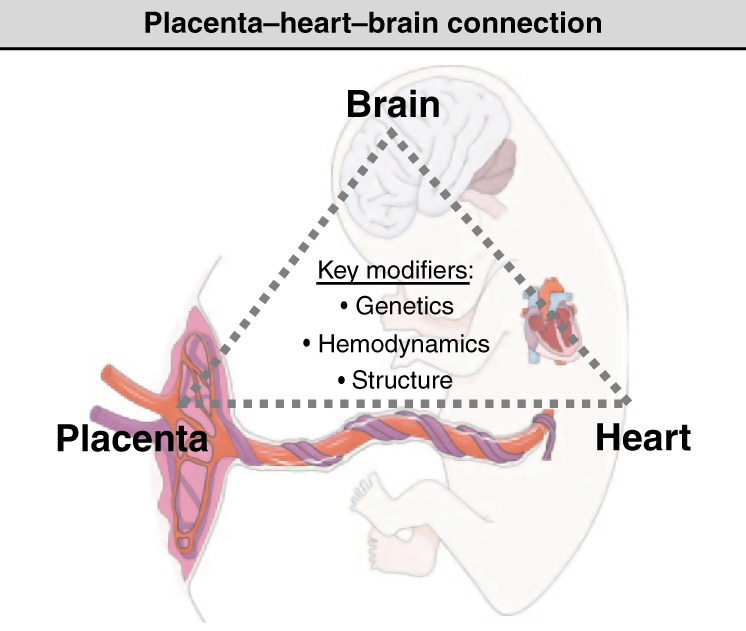
The placenta–heart–brain connection is modified by genetic/epigenetic, hemodynamic, and structural/microstructural influences. These represent key areas for future investigations in the field of neuroplacentology in CHD.

The gaps in our understanding of placental effects on brain development in patients with CHD involve several key areas: (1) differences in structure and function of the CHD placenta, (2) factors that impact the hemodynamic balance between the low resistance placental vascular bed and the higher resistance cerebral circulation, (3) interventions to improve placental function and protect brain development in utero, and (4) the role of genetic and epigenetic influences. These knowledge gaps underscore the three key modifiers in the placenta–heart–brain connection, which include genetics/epigenetics, hemodynamics, and organ structure and microstructure (Fig. 2). We propose future directions for both the clinical care and research into perinatal origins of neurodevelopmental impairments in those with CHD.

Our clinical protocol recommends obtaining a pathologic examination of the placenta in all pregnancies complicated by fetal CHD requiring interventions in the neonatal period. The yield is upwards of 80% in identifying pathologic lesions in the CHD placenta at our center (unpublished data), and strict adherence to the accepted Amsterdam classification system of placental pathology will allow direct comparisons between patients and across centers.^124^ Doppler ultrasound measures of umbilical artery and middle cerebral artery pulsatility indices should be considered in all pregnancies complicated by fetal CHD in order to risk-stratify this population in terms of likelihood of neurodevelopmental problems. Careful technique in measuring the pulsatility index of the middle cerebral artery is required for reliable data analysis. Postnatally, all neonates who will undergo surgical correction of CHD should ideally receive preoperative brain MRI, as recommended by experts in the field.^54,125^ Neurodevelopmental follow-up is paramount for these children and should be a routine part of their care, ideally in a setting familiar with their unique medical challenges. Providers should recognize the role of socioeconomic barriers to optimal neurodevelopment in children with CHD^126^ and facilitate identification and access to resources that will improve outcomes in vulnerable individuals.

From a research standpoint, future investigations will benefit from further development and validation of noninvasive functional placental imaging. Investigators should consider functional placental imaging at multiple timepoints in pregnancy to follow the trajectory of placental hemodynamics and its influence on fetal brain maturation, as single measurements can be misleading given the wide range of normal variation in most measures of placental size and perfusion.^7,90,127^ These longitudinal studies are limited by the high cost of MRI but are necessary to understand the timing of placental functional deficits and the specific effects on fetal brain development. The timing of disrupted brain development is essential to inform the optimal use of targeted interventions in these pregnancies. In fact, multiple clinical trials are currently underway treating fetuses with single-ventricle physiology CHD with maternal hyperoxygenation from second trimester to term (ClinicalTrials.gov NCT03136835, NCT02965638, NCT03147014). In addition to evaluation at multiple timepoints, research in CHD populations will benefit from multicenter aggregate data to allow for analyses based on physiologic groupings of CHD, like that being collected by the Cardiac Neurodevelopmental Outcome Collaborative Clinical Registry.^128^ Likewise, data from healthy control groups are paramount to making strong unbiased conclusions, and controls are commonly lacking in the CHD literature. Postpartum placental tissue examination should be included in all placental imaging studies, as tissue- and cellular-level data are required to explain the big picture imaging results and will allow us to begin to understand underlying mechanisms of perfusion abnormalities. Lastly, these studies, and all placental and fetal brain imaging studies in CHD, should include neurodevelopmental follow-up in order to determine the clinical significance of experimental results and interventions. This data is readily available at many centers where neurodevelopmental follow-up for children with CHD is routinely provided.

## Conclusion

We have presented evidence of impaired brain development in patients with CHD and outlined the potential for a prenatal influence. We have also described differences in the placenta of pregnancies complicated by CHD both in imaging studies and by histopathology. Taken together, these data suggest the likely contribution of the placenta to abnormal brain development in the CHD fetus. Future efforts to improve neurodevelopmental outcomes in CHD should focus on optimizing the intrauterine environment. Key areas for future research and improved clinical care in fetal CHD should focus on longitudinal assessments of both the placenta and fetal brain.

## References

[1] Reller MD, Strickland MJ, Riehle-Colarusso T, Mahle WT, Correa A (2008). Prevalence of congenital heart defects in metropolitan Atlanta, 1998–2005. J. Pediatr..

[2] Hoffman JI, Kaplan S (2002). The incidence of congenital heart disease. J. Am. Coll. Cardiol..

[3] Marino BS (2012). Neurodevelopmental outcomes in children with congenital heart disease: evaluation and management: a scientific statement from the American Heart Association. Circulation.

[4] Mussatto KA (2014). Risk and prevalence of developmental delay in young children with congenital heart disease. Pediatrics.

[5] Albalawi A (2017). Placental characteristics of fetuses with congenital heart disease. J. Ultrasound Med..

[6] Andescavage N (2015). 3-D volumetric MRI evaluation of the placenta in fetuses with complex congenital heart disease. Placenta.

[7] Zun Z, Zaharchuk G, Andescavage NN, Donofrio MT, Limperopoulos C (2017). Non-invasive placental perfusion imaging in pregnancies complicated by fetal heart disease using velocity-selective arterial spin labeled MRI. Sci. Rep..

[8] Kratimenos P, Penn AA (2019). Placental programming of neuropsychiatric disease. Pediatr. Res..

[9] Bonnin A (2011). A transient placental source of serotonin for the fetal forebrain. Nature.

[10] Knox K, Leuenberger D, Penn AA, Baker JC (2011). Global hormone profiling of murine placenta reveals Secretin expression. Placenta.

[11] Sun L (2015). Reduced fetal cerebral oxygen consumption is associated with smaller brain size in fetuses with congenital heart disease. Circulation.

[12] Lawley CM (2019). School-age developmental and educational outcomes following cardiac procedures in the first year of life: a population-based record linkage study. Pediatr. Cardiol..

[13] Shillingford AJ (2008). Inattention, hyperactivity, and school performance in a population of school-age children with complex congenital heart disease. Pediatrics.

[14] Do QN (2019). Texture analysis of magnetic resonance images of the human placenta throughout gestation: a feasibility study. PLoS ONE.

[15] Do, Q. N. et al. MRI of the placenta accreta spectrum (PAS) disorder: radiomics analysis correlates with surgical and pathological outcome. *J. Magn. Reson. Imaging* **51**, 936–946 (2020). 10.1002/jmri.26883 31397528

[16] Tsao PC (2017). Additive effect of congenital heart disease and early developmental disorders on attention-deficit/hyperactivity disorder and autism spectrum disorder: a nationwide population-based longitudinal study. Eur. Child Adolesc. Psychiatry.

[17] Wernovsky G (2000). Cognitive development after the Fontan operation. Circulation.

[18] Hovels-Gurich HH (2006). Long-term neurodevelopmental outcome and exercise capacity after corrective surgery for tetralogy of Fallot or ventricular septal defect in infancy. Ann. Thorac. Surg..

[19] Bellinger DC (1999). Developmental and neurological status of children at 4 years of age after heart surgery with hypothermic circulatory arrest or low-flow cardiopulmonary bypass. Circulation.

[20] Mahle WT (2000). Neurodevelopmental outcome and lifestyle assessment in school-aged and adolescent children with hypoplastic left heart syndrome. Pediatrics.

[21] Bellinger DC (2003). Neurodevelopmental status at eight years in children with dextro-transposition of the great arteries: the Boston Circulatory Arrest Trial. J. Thorac. Cardiovasc. Surg..

[22] van Rijen EH (2003). Psychosocial functioning of the adult with congenital heart disease: a 20-33 years follow-up. Eur. Heart J..

[23] Robson VK (2019). Longitudinal associations between neurodevelopment and psychosocial health status in patients with repaired D-transposition of the great arteries. J. Pediatr..

[24] Kassa AM, Dahl M, Strinnholm M, Engstrand, Lilja H (2020). Attention difficulties and physical dysfunction common in children with complex congenital malformations: a study of preschool children with VACTERL association. Acta Paediatr..

[25] Gaynor JW (2007). Patient characteristics are important determinants of neurodevelopmental outcome at one year of age after neonatal and infant cardiac surgery. J. Thorac. Cardiovasc. Surg..

[26] Gaynor JW (2009). Apolipoprotein E genotype modifies the risk of behavior problems after infant cardiac surgery. Pediatrics.

[27] Dunbar-Masterson C (2001). General health status of children with D-transposition of the great arteries after the arterial switch operation. Circulation.

[28] Reich B (2019). Neurodevelopmental outcome in hypoplastic left heart syndrome after hybrid procedure. Transl. Pediatr..

[29] Bellinger DC (2009). Behaviour at eight years in children with surgically corrected transposition: the Boston Circulatory Arrest Trial. Cardiol. Young.

[30] Pike NA (2016). Predictors of memory deficits in adolescents and young adults with congenital heart disease compared to healthy controls. Front. Pediatr..

[31] Lane DA, Lip GY, Millane TA (2002). Quality of life in adults with congenital heart disease. Heart.

[32] Horner T, Liberthson R, Jellinek MS (2000). Psychosocial profile of adults with complex congenital heart disease. Mayo Clin. Proc..

[33] Brandhagen DJ, Feldt RH, Williams DE (1991). Long-term psychologic implications of congenital heart disease: a 25-year follow-up. Mayo Clin. Proc..

[34] Wernovsky, G. Factors influencing neurodevelopmental outcomes in neonates with CHD: deciphering the modifiable & the unmodifiable towards understanding and optimizing neurodevelopmental outcomes. Neonatal Heart Society, NeoHeart 2020: a global virtual event. https://neoheartsociety.org/conference2020/ (2020).

[35] Glessner JT (2014). Increased frequency of de novo copy number variants in congenital heart disease by integrative analysis of single nucleotide polymorphism array and exome sequence data. Circ. Res..

[36] Zaidi S, Brueckner M (2017). Genetics and genomics of congenital heart disease. Circ. Res..

[37] Jin SC (2017). Contribution of rare inherited and de novo variants in 2,871 congenital heart disease probands. Nat. Genet..

[38] Lynch JM, Gaynor JW, Licht DJ (2018). Brain injury during transition in the newborn with congenital heart disease: hazards of the preoperative period. Semin. Pediatr. Neurol..

[39] Mebius MJ (2016). Cerebral oxygen saturation during the first 72h after birth in infants diagnosed prenatally with congenital heart disease. Early Hum. Dev..

[40] Andropoulos DB (2014). The association between brain injury, perioperative anesthetic exposure, and 12-month neurodevelopmental outcomes after neonatal cardiac surgery: a retrospective cohort study. Paediatr. Anaesth..

[41] Diaz LK (2016). Increasing cumulative exposure to volatile anesthetic agents is associated with poorer neurodevelopmental outcomes in children with hypoplastic left heart syndrome. J. Thorac. Cardiovasc. Surg..

[42] Hoffman GM, Brosig CL, Bear LM, Tweddell JS, Mussatto KA (2016). Effect of intercurrent operation and cerebral oxygenation on developmental trajectory in congenital heart disease. Ann. Thorac. Surg..

[43] Everett AD (2020). Association of neurodevelopmental outcomes with environmental exposure to cyclohexanone during neonatal congenital cardiac operations: a secondary analysis of a randomized clinical trial. JAMA Netw. Open.

[44] McQuillen PS (2007). Temporal and anatomic risk profile of brain injury with neonatal repair of congenital heart defects. Stroke.

[45] Beca J (2013). New white matter brain injury after infant heart surgery is associated with diagnostic group and the use of circulatory arrest. Circulation.

[46] Sananes R (2012). Neurodevelopmental outcomes after open heart operations before 3 months of age. Ann. Thorac. Surg..

[47] Wolfe KR, Brinton J, Di Maria MV, Meier M, Liptzin DR (2019). Oxygen saturations and neurodevelopmental outcomes in single ventricle heart disease. Pediatr. Pulmonol..

[48] Walker K (2015). Developmental outcomes at 3 years of age following major non-cardiac and cardiac surgery in term infants: a population-based study. J. Paediatr. Child Health.

[49] Kobayashi Y (2020). Association between surgical procedures under general anesthesia in infancy and developmental outcomes at 1 year: the Japan Environment and Children’s Study. Environ. Health Prev. Med..

[50] Zeng S (2015). Volume of intracranial structures on three-dimensional ultrasound in fetuses with congenital heart disease. Ultrasound Obstet. Gynecol..

[51] von Rhein M (2015). Severe congenital heart defects are associated with global reduction of neonatal brain volumes. J. Pediatr..

[52] Ortinau C (2012). Regional alterations in cerebral growth exist preoperatively in infants with congenital heart disease. J. Thorac. Cardiovasc. Surg..

[53] Miller SP (2004). Preoperative brain injury in newborns with transposition of the great arteries. Ann. Thorac. Surg..

[54] Miller SP (2007). Abnormal brain development in newborns with congenital heart disease. N. Engl. J. Med..

[55] Kelly CJ (2019). Abnormal microstructural development of the cerebral cortex in neonates with congenital heart disease is associated with impaired cerebral oxygen delivery. J. Am. Heart Assoc..

[56] Kelly CJ (2019). Neuroimaging findings in newborns with congenital heart disease prior to surgery: an observational study. Arch. Dis. Child.

[57] Guo T (2019). White matter injury in term neonates with congenital heart diseases: topology & comparison with preterm newborns. Neuroimage.

[58] Glauser TA, Rorke LB, Weinberg PM, Clancy RR (1990). Congenital brain anomalies associated with the hypoplastic left heart syndrome. Pediatrics.

[59] Glauser TA, Rorke LB, Weinberg PM, Clancy RR (1990). Acquired neuropathologic lesions associated with the hypoplastic left heart syndrome. Pediatrics.

[60] De Asis-Cruz J, Donofrio MT, Vezina G, Limperopoulos C (2018). Aberrant brain functional connectivity in newborns with congenital heart disease before cardiac surgery. Neuroimage Clin..

[61] Claessens NHP (2019). Brain injury in infants with critical congenital heart disease: insights from two clinical cohorts with different practice approaches. J. Pediatr..

[62] Claessens NH (2016). Delayed cortical gray matter development in neonates with severe congenital heart disease. Pediatr. Res..

[63] Khalil A (2014). Brain abnormalities and neurodevelopmental delay in congenital heart disease: systematic review and meta-analysis. Ultrasound Obstet. Gynecol..

[64] Rollins CK (2017). White matter volume predicts language development in congenital heart disease. J. Pediatr..

[65] Peyvandi S (2018). Neonatal brain injury and timing of neurodevelopmental assessment in patients with congenital heart disease. J. Am. Coll. Cardiol..

[66] Licht DJ (2009). Brain maturation is delayed in infants with complex congenital heart defects. J. Thorac. Cardiovasc. Surg..

[67] Ni Bhroin M (2020). Reduced structural connectivity in cortico-striatal-thalamic network in neonates with congenital heart disease. Neuroimage Clin..

[68] Jorgensen DES (2018). Longitudinal brain and body growth in fetuses with and without transposition of the great arteries: quantitative volumetric magnetic resonance imaging study. Circulation.

[69] Limperopoulos C (2010). Brain volume and metabolism in fetuses with congenital heart disease: evaluation with quantitative magnetic resonance imaging and spectroscopy. Circulation.

[70] Olshaker H (2018). Volumetric brain MRI study in fetuses with congenital heart disease. AJNR Am. J. Neuroradiol..

[71] Welling MS (2020). Growth trajectories of the human fetal brain in healthy and complicated pregnancies and associations with neurodevelopmental outcome in the early life course. Early Hum. Dev..

[72] Clouchoux C (2012). Quantitative in vivo MRI measurement of cortical development in the fetus. Brain Struct. Funct..

[73] Paladini, D. et al. Frontal lobe growth is impaired in fetuses with congenital heart disease. *Ultrasound Obstet. Gynecol.*10.1002/uog.22127 (2020). 10.1002/uog.22127 32573836

[74] Rajagopalan V (2018). Fetuses with single ventricle congenital heart disease manifest impairment of regional brain growth. Prenat. Diagn..

[75] Jansen FA (2016). Head growth in fetuses with isolated congenital heart defects: lack of influence of aortic arch flow and ascending aorta oxygen saturation. Ultrasound Obstet. Gynecol..

[76] Yamamoto Y (2013). Severe left heart obstruction with retrograde arch flow influences fetal cerebral and placental blood flow. Ultrasound Obstet. Gynecol..

[77] Lauridsen MH (2017). Cerebral oxygenation measurements by magnetic resonance imaging in fetuses with and without heart defects. Circ. Cardiovasc. Imaging.

[78] Donofrio MT, Duplessis AJ, Limperopoulos C (2011). Impact of congenital heart disease on fetal brain development and injury. Curr. Opin. Pediatr..

[79] Mallard EC, Rees S, Stringer M, Cock ML, Harding R (1998). Effects of chronic placental insufficiency on brain development in fetal sheep. Pediatr. Res..

[80] Zeng S (2015). Assessment by three-dimensional power Doppler ultrasound of cerebral blood flow perfusion in fetuses with congenital heart disease. Ultrasound Obstet. Gynecol..

[81] Donofrio MT (2003). Autoregulation of cerebral blood flow in fetuses with congenital heart disease: the brain sparing effect. Pediatr. Cardiol..

[82] Kaltman JR, Di H, Tian Z, Rychik J (2005). Impact of congenital heart disease on cerebrovascular blood flow dynamics in the fetus. Ultrasound Obstet. Gynecol..

[83] Szwast A, Tian Z, McCann M, Donaghue D, Rychik J (2010). Vasoreactive response to maternal hyperoxygenation in the fetus with hypoplastic left heart syndrome. Circ. Cardiovasc. Imaging.

[84] Szwast A, Tian Z, McCann M, Soffer D, Rychik J (2012). Comparative analysis of cerebrovascular resistance in fetuses with single-ventricle congenital heart disease. Ultrasound Obstet. Gynecol..

[85] Alves de Alencar Rocha AK (2017). Early- versus late-onset fetal growth restriction differentially affects the development of the fetal sheep brain. Dev. Neurosci..

[86] Back SA (2002). Selective vulnerability of late oligodendrocyte progenitors to hypoxia-ischemia. J. Neurosci..

[87] Buser JR (2012). Arrested preoligodendrocyte maturation contributes to myelination failure in premature infants. Ann. Neurol..

[88] Gaynor JW (2018). The impact of the maternal-foetal environment on outcomes of surgery for congenital heart disease in neonates. Eur. J. Cardiothorac. Surg..

[89] Jones HN (2015). Hypoplastic left heart syndrome is associated with structural and vascular placental abnormalities and leptin dysregulation. Placenta.

[90] Leon RL, Li KT, Brown BP (2018). A retrospective segmentation analysis of placental volume by magnetic resonance imaging from first trimester to term gestation. Pediatr. Radiol..

[91] Langhoff L (2017). Placental growth during normal pregnancy - a magnetic resonance imaging study. Gynecol. Obstet. Investig..

[92] You, W. et al. Hemodynamic responses of the placenta and brain to maternal hyperoxia in fetuses with congenital heart disease by using blood oxygen-level dependent MRI. *Radiology* **294**, 141–148 (2020). 10.1148/radiol.2019190751 PMC6939742 31687920

[93] Szwast A, Putt M, Gaynor JW, Licht DJ, Rychik J (2018). Cerebrovascular response to maternal hyperoxygenation in fetuses with hypoplastic left heart syndrome depends on gestational age and baseline cerebrovascular resistance. Ultrasound Obstet. Gynecol..

[94] Ho, D. Y. et al. Mid-gestational fetal placental blood flow is diminished in the fetus with congenital heart disease. *Prenat. Diagn*. **40**, 1432–1438 (2020). 10.1002/pd.5791 32673414

[95] Binder J (2020). Evidence for uteroplacental malperfusion in fetuses with major congenital heart defects. PLoS ONE.

[96] Rychik J (2018). Characterization of the placenta in the newborn with congenital heart disease: distinctions based on type of cardiac malformation. Pediatr. Cardiol..

[97] Hassink SG (1997). Placental leptin: an important new growth factor in intrauterine and neonatal development?. Pediatrics.

[98] Courtney J (2020). Abnormalities of placental development and function are associated with the different fetal growth patterns of hypoplastic left heart syndrome and transposition of the great arteries. Placenta.

[99] Maslen CL (2018). Recent advances in placenta-heart interactions. Front. Physiol..

[100] Cohen JA, Rychik J, Savla JJ (2021). The placenta as the window to congenital heart disease. Curr. Opin. Cardiol..

[101] Courtney JA, Cnota JF, Jones HN (2018). The role of abnormal placentation in congenital heart disease; cause, correlate, or consequence?. Front. Physiol..

[102] Russell MW (2019). Effect of parental origin of damaging variants in pro-angiogenic genes on fetal growth in patients with congenital heart defects: data and analyses. Data Brief..

[103] Russell MW (2019). Damaging variants in proangiogenic genes impair growth in fetuses with cardiac defects. J. Pediatr..

[104] Carmeliet P (1996). Abnormal blood vessel development and lethality in embryos lacking a single VEGF allele. Nature.

[105] Miquerol L, Langille BL, Nagy A (2000). Embryonic development is disrupted by modest increases in vascular endothelial growth factor gene expression. Development.

[106] Kawasaki T (1999). A requirement for neuropilin-1 in embryonic vessel formation. Development.

[107] Bedard T (2013). Folic acid fortification and the birth prevalence of congenital heart defect cases in Alberta, Canada. Birth Defects Res. A Clin. Mol. Teratol..

[108] Czeizel AE, Dudas I, Vereczkey A, Banhidy F (2013). Folate deficiency and folic acid supplementation: the prevention of neural-tube defects and congenital heart defects. Nutrients.

[109] Feng Y (2015). Maternal folic acid supplementation and the risk of congenital heart defects in offspring: a meta-analysis of epidemiological observational studies. Sci. Rep..

[110] Ionescu-Ittu R, Marelli AJ, Mackie AS, Pilote L (2009). Prevalence of severe congenital heart disease after folic acid fortification of grain products: time trend analysis in Quebec, Canada. BMJ.

[111] Li X (2013). The association between periconceptional folic acid supplementation and congenital heart defects: a case-control study in China. Prev. Med..

[112] Qu Y (2020). First-trimester maternal folic acid supplementation reduced risks of severe and most congenital heart diseases in offspring: a large case-control study. J. Am. Heart Assoc..

[113] Hobbs CA, Cleves MA, Melnyk S, Zhao W, James SJ (2005). Congenital heart defects and abnormal maternal biomarkers of methionine and homocysteine metabolism. Am. J. Clin. Nutr..

[114] Kapusta L (1999). Congenital heart defects and maternal derangement of homocysteine metabolism. J. Pediatr..

[115] Sutton M (2011). Maternal folate, vitamin B12 and homocysteine levels in pregnancies affected by congenital malformations other than neural tube defects. Birth Defects Res. A Clin. Mol. Teratol..

[116] Frosst P (1995). A candidate genetic risk factor for vascular disease: a common mutation in methylenetetrahydrofolate reductase. Nat. Genet..

[117] Wang D (2017). Lower circulating folate induced by a fidgetin intronic variant is associated with reduced congenital heart disease susceptibility. Circulation.

[118] Wang J (2014). A genetic variant in vitamin B12 metabolic genes that reduces the risk of congenital heart disease in Han Chinese populations. PLoS ONE.

[119] Xuan C (2014). Association between MTHFR polymorphisms and congenital heart disease: a meta-analysis based on 9,329 cases and 15,076 controls. Sci. Rep..

[120] Durbin MD, O’Kane J, Lorentz S, Firulli AB, Ware SM (2020). SHROOM3 is downstream of the planar cell polarity pathway and loss-of-function results in congenital heart defects. Dev. Biol..

[121] Henderson DJ, Chaudhry B (2011). Getting to the heart of planar cell polarity signaling. Birth Defects Res. A Clin. Mol. Teratol..

[122] Wu G, Ge J, Huang X, Hua Y, Mu D (2011). Planar cell polarity signaling pathway in congenital heart diseases. J. Biomed. Biotechnol..

[123] Hakanen J, Ruiz-Reig N, Tissir F (2019). Linking cell polarity to cortical development and malformations. Front. Cell. Neurosci..

[124] Khong TY (2016). Sampling and definitions of placental lesions: Amsterdam Placental Workshop Group Consensus Statement. Arch. Pathol. Lab. Med..

[125] Peyvandi S, Latal B, Miller SP, McQuillen PS (2019). The neonatal brain in critical congenital heart disease: insights and future directions. Neuroimage.

[126] Favilla, E. et al. Early evaluation and the effect of socioeconomic factors on neurodevelopment in infants with tetralogy of Fallot. *Pediatr. Cardiol*. **42**, 643–653 (2021). 10.1007/s00246-020-02525-6 PMC7990815 33533966

[127] Anblagan D (2013). Maternal smoking during pregnancy and fetal organ growth: a magnetic resonance imaging study. PLoS ONE.

[128] Cardiac Neurodevelopmental Outcome Collaborative. https://www.cardiacneuro.org/join/ (2021).

